# Supplementary material for: Enhancing Genetic Medicine: Rapid and Cost-Effective Molecular Diagnosis for a GJB2 Founder Mutation for Hearing Impairment in Ghana
Source: Genes (Basel). 2020 Jan 27;11(2):132. doi: 10.3390/genes11020132 (PMC7074138; doi:10.3390/genes11020132)
Supplement: Supplementary file 1 [file genes-11-00132-s001.pdf]

Supplementary Materials

Table S1: Primer sequencing for *GJB2* and *GJB6* coding region amplification

**Gene**	**Primer**	**Primer sequence**	**Product size**
GJB2	F4	5′ -GCTTACCCAGACTCAGAGAAG-3′	900
	R1	5′-CTTAATCTAACAACTGGGCAATGC-3′	
GJB6	CDF	5′-TTGGCTTCAGTATGTAATATCACC-3′	990
	CDR	5′-TCATTTACAAACTCTTCAGGCTACAG-3′	

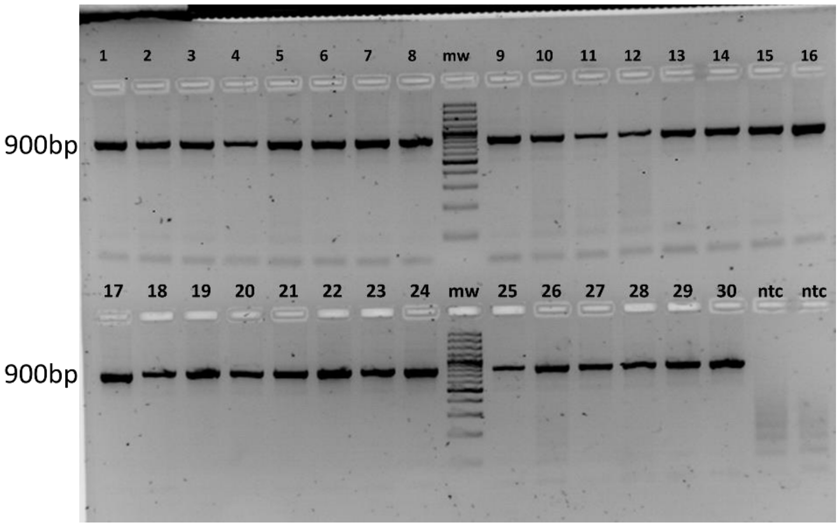

Figure S1: Representative agarose gel picture of *GJB2* exon 2 PCR products. The numbers 1 to 30 are the samples and "mw" and "ntc" are the molecular weight marker and the non-template controls respectively.
